# Investigation of Surface Roughness in Incremental Sheet Forming of Conical Drawpieces from Pure Titanium Sheets

**DOI:** 10.3390/ma15124278

**Published:** 2022-06-16

**Authors:** Tomasz Trzepieciński, Marcin Szpunar, Andrzej Dzierwa, Krzysztof Żaba

**Affiliations:** 1 Department of Manufacturing and Production Engineering, Faculty of Mechanical Engineering and Aeronautics, Rzeszow University of Technology, al. Powst. Warszawy 8, 35-959 Rzeszów, Poland; adzierwa@prz.edu.pl; 2 Doctoral School of Engineering and Technical Sciences at the Rzeszow University of Technology, Rzeszow University of Technology, al. Powst. Warszawy 12, 35-959 Rzeszów, Poland; d547@stud.prz.edu.pl; 3 Department of Metal Working and Physical Metallurgy of Non-Ferrous Metals, Faculty of Non-Ferrous Metals, University of Science and Technology, al. Adama Mickiewicza 30, 30-059 Cracow, Poland; krzyzaba@agh.edu.pl

**Keywords:** incremental sheet forming, sheet metals, single point incremental forming, SPIF

## Abstract

The article presents the results of the analysis of the influence of incremental sheet forming process parameters on surface roughness measured on both sides of conical drawpieces made from pure titanium Grade 2 sheets. The experimental plan was created on the basis of a central composite design. The study assumed the variability of feed rate, spindle speed, and incremental step size in the following range: 500–2000 mm/min, 0–600 rpm, and 0.1–0.5 mm, respectively. Two strategies differing in the direction of the tool rotation in relation to the feed direction were also analysed. Analysis of variance is performed to understand the adequacy of the proposed model and the influence of the input parameters on the specific roughness parameter. The sensitivity of the process parameter on the selected surface roughness parameters was assessed using artificial neural networks. It was found that the change in the surface roughness of the inner surface of the drawpiece is not related to the change of surface roughness of the outer side. The morphology of the outer surface of the draw pieces was uniform with a much greater profile height than the inner surface that had interacted with the tool. Taking into account the outer surface of the drawpiece, the direction of tool rotation is also most closely correlated with the parameters Sa, Sz, and Sku. Step size and feed rate provide the highest information capacity in relation to skewness and kurtosis of the inner surface of the drawpiece.

## 1. Introduction

There is a growing market demand for greater speed and flexibility in the development of new products, with an increasing tendency towards individualisation. The development of new forming methods is particularly desirable in the production of prototypes and short-run production. The manufacturing of products in small series using the conventional methods of sheet metal forming (SMF), i.e., stretch forming or deep drawing, requires the production of costly dies with a shape adapted to the shape of a specific product. To meet these requirements, the small- and ultra-small-lot manufacturing industry is increasingly turning to incremental sheet forming (ISF) processes. ISF consists in deforming the workpiece by means of gradual local deformation of the sheet metal using a pin-type tool [1,2].

In general, ISF methods can be divided into single-point incremental forming (SPIF) and two-point incremental forming (TPIF) with a counter tool or partial die [3]. In the simplest variant, a machine that is numerically controlled in at least three axes is required for the forming process. SPIF can be carried out on milling machines (movable tool, stationary workpiece) or a turning machine (stationary tool, rotatable workpiece) [4]. The edge of the workpiece is usually fixed to avoid any movement. The forming process consists in moving a rotating or non-rotating spindle along a three-dimensional path that gradually sinks towards the height of the workpiece [5]. Among the many tool path strategies, two are the most common. In the first case, the tool moves downwards to the final position along a continuous path with a linear vertical pitch. In the second strategy, the tool moves along multi-step paths with z-level contouring. Alves de Sousa et al. [6] introduced a new concept of ISF equipment that possesses six-degrees-of-freedom for the tool, for the sake of improved flexibility in terms of an extra stiffness provided by a parallel kinematics scheme. The increase in the flexibility of the SPIF process by fitting to a given geometry, increasing the maximum part size, and reducing material waste was analysed by Afonso et al. [7].

Although this article focuses on SPIF by means of a metallic spindle, ISF variants carried out using an electromagnetic pulse [8,9] or by means of a high-pressure water jet [10,11] are also known. Hot forming methods [12,13] have been developed for forming hard-to-deform materials in cold forming conditions. Conventional SMF processes require larger quantities of lubricant than is allowed for environmental reasons. To assure the minimisation of the lubricant used, tools with thin layered tungsten carbide coating can be used [14].

Despite the many advantages of SPIF, such as greater forming limits and lower forming forces compared to conventional deep drawing and quick modification of design changes in components, this technology has several limitations [15,16]. First of all, the drawpieces show a relatively large springback; incorrectly selected input parameters may result in unsatisfactory roughness of the inner surface of the drawpiece. In areas with small rounding radii, a reduction in the geometric accuracy of the products is observed [17,18].

The accurate prediction of the geometry of the SPIFed component is a key issue for ensuring high-quality products. According to Pepelnjak et al. [19] and Micari et al. [20], the geometric errors of the final components obtained by the SPIF can be divided into three categories. The first type of geometric error of drawpieces is a protrusion or concave curvature occurring on the undeformed bottom of the part also known as the ‘pillow effect’. This error is particularly emphasised on drawpieces with a flat bottom and is the main reason for the geometrical inaccuracy of the ISF process [21]. The second source of the geometrical inaccuracy is elastic deformation of the sheet metal (springback) caused by a drop in the stress when the drawpiece is unloaded. Elastic springback may occur after the release of the drawpiece from the forming device and after the tool is released. The residual stresses arising during the cyclic loading and unloading of the SPIFed component increase the springback effect and geometric inaccuracy [22]. The third type of geometric inaccuracy of the incrementally formed components arises at the beginning of the ISF process. Discrepancies between the actual part geometry and the desired one are caused by an undesirable bending of the clamped sheet along the edge of the main base. This source of the geometrical inaccuracy may be limited to using a simple backing plate or applying for the rigid support next to the forming zone [19].

The use of suitable lubricants is of great importance in obtaining the desired surface quality of components. Moreover, the use of lubricants is essential at the interface between tool and workpiece in order to improve heat distribution and reduce wear and friction [23,24]. Şen et al. [25] applied the minimum quantity lubrication (MQL) technique for the SPIF process to investigate its effectiveness in SPIF processes. Sheet metal has been formed with the SPIF process by using vegetable-based oils and a paste lubricant. It was found that the surface quality can be improved by 14.60% with the MQL-assisted SPIF process. Moreover, an increase in pressure has greatly increased the surface quality and dimensional accuracy of SPIFed components. Azevedo et al. [26] studied the effect of the type of lubricant in SPIF on the surface quality of components made of DP780 steel and AA1050 aluminium. Petroleum and mineral oils, as well as pastes, were used as lubricants. It was found that the greater the hardness of the material to form, the lower the necessary viscosity of the lubricant. The effect of the process parameters on surface roughness in SPIF of AA1050 material using a dummy sheet was studied by Sisodia and Kumar [27]. Process parameters such as dummy sheet thickness, wall angle, step size, and tool size was found significant in influencing the mean roughness Ra of the formed part. Furthermore, with the increase in dummy sheet thickness, the mean roughness Ra value decreases. Skjødt et al. [28] have used a dummy sheet to improve the part finishing of components specially made of soft aluminium sheets. The use of a dummy sheet setup improves surface roughness, eliminates wear, and causes a small reduction in formability. To improve the wall thickness distribution of the HC380LA steel part resulting from TPIF, the rolling blank holder (TPIF-RBH) method was proposed by Şen et al. [29]. Based on the gray relational analysis it was found that tool diameter has a great impact on surface roughness. It was observed that the surface quality increased with the increasing tool diameter.

The surface roughness of the components is one of the biggest challenges facing technologists who design SPIF processes. The purpose of most studies is to determine the effect of the process parameters on the quality of the surface that was in contact with the tool. Various approaches are taken to optimise the surface roughness by controlling four machining parameters, i.e., tool rotational speed, tool size, feed rate and step size, and the use of an appropriate lubricant [30]. Sisodia and Kumar [27] formed conical drawpieces from AA1050 aluminium alloy sheets. With the help of analysis of variance (ANOVA), they found that step size and tool size are significant parameters influencing the mean roughness Ra of the formed part. On the other hand, the feed rate is found to be non-significant. Najm and Paniti [31] used artificial neural networks (ANNs) to predict the Ra and Rz roughness parameters of the AlMn1Mg1 aluminium alloy frustum drawpieces SPIFed using tools from various materials. It was found that the mean roughness Ra of the tool surface and tool materials play a significantly important role in affecting the sheet surface roughness. The Rz parameter of the drawpiece surface strongly depends on the ten-point mean roughness Rz of the tool. In another work [32], the authors found that the best surface roughness can be achieved with the fastest tool movement using the smallest incremental depth and the biggest forming tool. A decrement in step depth, feed rate, and tool rotational speed resulted in decreasing surface roughness of EN AW-6063 drawpieces [33]. Furthermore, the lubrication conditions ensure less surface roughness compared to dry friction. Rattanachan and Chungchoo [34] used the 2k-p factorial experimental design to investigate the interaction between step depth, feed rate, tool radius, and mean surface roughness of DIN 1.0037 steel components. It was found that reducing feed rate and tool rotational speed reduced inner mean surface roughness. Increasing the depth step and feed rate decreased the inner surface roughness. Dakhli et al. [35] applied the response surface methodology and Taguchi grey relational analysis to obtain an optimal set of input parameters with respect to the surface roughness of AA1050 and DC01 frustums. Based on the ANOVA analysis, the lubricant and sheet material are the most significant factors that affect surface roughness. Singh [36] studied the effects of several parameters at different levels of surface roughness of AA2014 aluminium alloy sheets. It was concluded that tool path, step size, and feed rate have little effect on surface roughness. Lubrication conditions and tool rotational speed significantly affect the surface roughness of SPIFed drawpieces. Oraon and Sharma [37] used ANNs to predict the mean surface roughness of Cu67Zn33 components. The graphite lubricant greatly affected the Ra-value. The Ra-value predicted by ANNs was found to be quite close to the measured Ra, confirming the applicability of ANNs to optimising the surface roughness in SPIF. The significant effects of the tool tip radius and lubricants on surface roughness were observed by Oleksik et al. [38] during the forming of medical implants from Ti-6Al-4V titanium sheets. Mulay et al. [39] observed that the most significant parameter affecting the surface roughness of AA5754 H22 aluminium alloy drawpieces is step size, followed by feed rate. The mean surface roughness increased with increasing step size. Radu [40] concluded that the tool radius and step size have a significant effect on the surface roughness of pyramidal frustums from DC01 steel sheets processed by SPIF. Higher tool end radius along with high tool rotational speed enhanced the surface quality of SPIFed parts made of AA1050 aluminium alloy sheets [41]. The experimental investigations of SPIF of a stainless steel denture framework in relation to surface quality show a significant influence of step size on surface roughness [42]. Mean surface roughness increased as step size increased.

In a previous paper [17], the authors used the optimal SPIF parameters in forming the truncated cones from commercially pure titanium Grade 2 sheet metals in order to minimise the maximum of both the in-plane and axial components. Response surface methodology was used to find relations between process parameters (tool feed rate, step size, and spindle speed) and the 10-point peak–valley surface roughness Rz parameter measured at the inner side of the drawpieces. It was found that drawpieces formed with high values of spindle speed showed poor surface qualities. A review of the literature [2] on SPIF of titanium and titanium alloy sheets showed that there are insufficient investigations into the synergistic effect of rotational speed and tool rotation direction on the surface roughness of SPIFed drawpieces. In another previous paper, [43], the authors applied a split-plot I-optimal design to optimise combined oil-based and friction stir rotation-assisted heating in SPIF of Ti-6Al-4V sheets. It was concluded that step size is the most significant factor that affects the in-plane forming force. Moreover, the step size is the most significant factor that affects the axial SPIF force, followed by feed rate. The effects of SPIF parameters on the surface roughness parameters of the outer and inner surfaces of drawpieces were not examined. The influence of some SPIF parameters on the surface roughness of formed elements is sometimes contradictory and sometimes debatable. Ham [44] found that step size affects surface roughness while Micari et al. [45] concluded the contrary. Hamilton and Jeswiet [46] suggested that high feed rates are preferable to limit the manufacturing time; however, Strano [47] and Tanaka et al. [48] concluded that a slower feed rate has a positive effect on formability and surface quality and so further investigations are necessary. Based on this review of the literature, it can be concluded that the majority of authors focus on the optimisation of input parameters in such a way as to obtain the lowest surface roughness on the side of the drawpiece where the spindle touches the sheet metal. Contrary to results from the literature, surface roughness in both inner and outer surfaces was analysed in this article. Meanwhile, the opposite surface of the drawpieces also changes significantly, mainly due to the ‘orange peel’ effect, and its quality also needs to be controlled. One of the justifications is to prepare the surface for painting, which must have the appropriate roughness. In this article, a 3D analysis was performed of the surface topography of the inner and outer surfaces of conical drawpieces SPIFed from Grade 2 titanium sheets. In addition, the research took into account the effect of different directions of tool rotation in relation to the direction of feed on the surface roughness parameters evaluated over the complete 3D surface. No similar research has been found for SPIF of Grade 2 titanium sheets. Contrary to results from the literature, where in practice the only mean roughness Ra [49,50,51,52,53,54,55] or two surface roughness parameters Ra and Rz [31,56,57] or Sa and Sz [58] are considered, six surface roughness parameters (Sa, Sz, Ssk, Sku, Sdq, and Spk) were analysed in this article and their significance was determined using ANNs. The experimental plan was created on the basis of central composite design (CCD). ANOVA is performed to understand the adequacy of the proposed model and the influence of the input parameters on the specific roughness parameter.

## 2. Materials and Methods

### 2.1. Material

A commercially pure (CP) Grade 2 titanium in 0.4-mm-thick sheets was used as a test material. CP Grade 2 titanium is a hexagonal allotropic α-state material structure and is the most widely used titanium grade in research studies. This grade combines good corrosion resistance, good cold formability, and weldability. It shows a favourable ratio between ductility and strength. It is commonly used in applications where excellent corrosion resistance and low density-to-strength ratio are demanded. Such assets give a potential for application in automotive, aerospace, biomedical, subsea, and marine equipment, as well as chemical processing industries. Table 1 presents the chemical composition of the test material delivered by the manufacturer. Mechanical parameters (Table 2) were determined using a uniaxial tensile test at room temperature according to ISO 6892-1:2016 [59]. The following parameters were determined: yield stress (YS), ultimate tensile stress (UTS), strain hardening coefficient (SHC), and strain hardening exponent (SHE).

### 2.2. Experimental Setup

**Figure 1:**
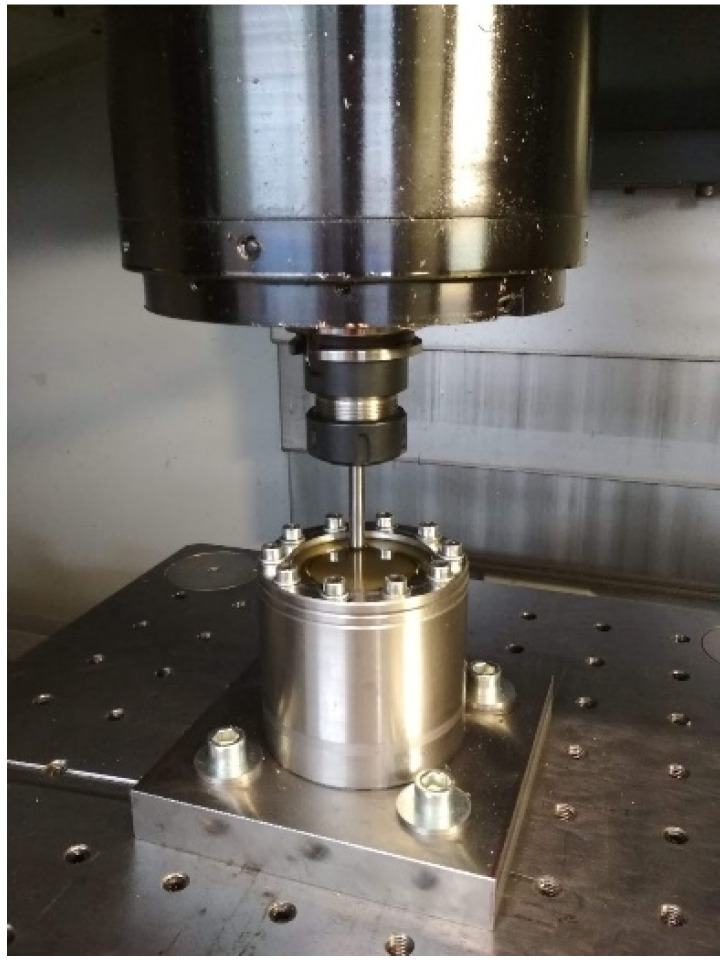
Forming device mounted inside CNC milling machine workspace.

The truncated cone geometry was determined as a specimen shape to be formed on a 3-axis computerized numerical control (CNC) PS95 milling machine. Special design equipment dedicated to the incremental forming process was mounted in the working space of the machine (Figure 1). Circular ∅100 mm blanks were cut from the sheet metal and clamped inside the forming device by tightening with screws at 10 nm torque.

A forming punch made of ∅8 mm sintered tungsten carbide rod rounded with a 4 mm radius was applied. The selection of the radius of tool is crucial to ensure the overall process window of SPIF. An excessively small radius of tool can push the process from a regular forming state to an irregular. In this condition, the tool plows the sheet thereby squeezing out the material from the tool/sheet interface and causing a premature failure [60]. On the other hand, the formability reduces as the curvature radius decreases [61]. Too large a tool radius creates excessive friction and degrades the surface quality of the drawpiece. Moreover, Hirt et al. [62] reported that the formability decreases as the tool radius increases. Taking the above-mentioned phenomena the tool radius was determined in preliminary experimental studies which results are published in [17].

**Figure 2:**
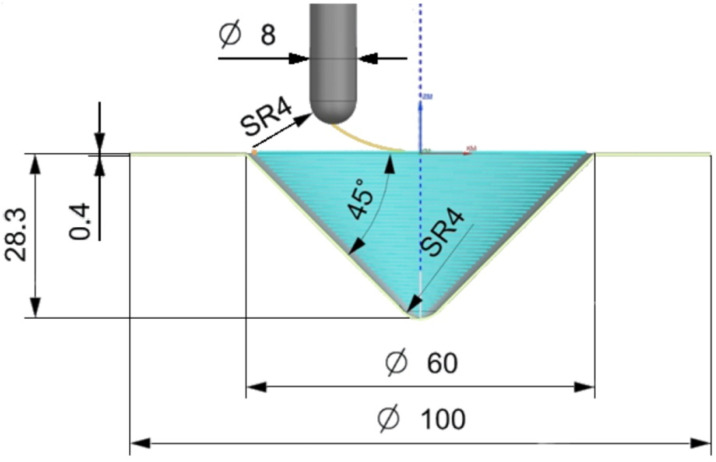
Drawpiece geometry and tool path generated in NX Siemens PLM.

The sample wall angle was 45°, which allowed the forming of a 28.3 mm height truncated cone drawpiece starting from a base diameter ∅60 mm (Figure 2). The tool trajectory was generated using an NX Siemens CAM (Siemens, Munich, Germany) version 1938. A spiral path was applied with a pitch equal to step size. As lubricant, 10W-40 semi-synthetic oil (Castrol Ltd., Liverpool, UK) was applied.

### 2.3. Forming Forces

Both the in-plane and axial components of SPIF during forming of the drawpieces from commercially pure titanium Grade 2 sheet metals were analysed in the previous paper [17] of the authors. The horizontal (*x*- and *y*-axes) and axial (*z*-axis) forces occurring during the incremental forming were measured by a high-accuracy piezoelectric dynamometer Kistler. Both the horizontal and the axial components of the SPIF force are directly related to the increase in step size, as also shown by Uheida et al. [63] and Petek et al. [64]. Furthermore, the spindle speed has no direct influence on the forming force components but has a strong influence on the surface roughness of the drawpiece. The axial force has a significant impact on material formability [65]. A major SPIF parameter affecting the in-plane force is the step size. The feed rate does not affect in-plane force, as also observed by Özgen et al. [66].

### 2.4. ANOVA and Central Composite Design

The central composite design was created to establish the effect of significant input parameters to surface roughness quality inside and outside the formed drawpiece. As input factors, spindle speed n, feed rate f, and step size a_p_ were selected. The direction of spindle rotation in relation to feed direction was also considered. Maximum and minimum levels of the experiment were obtained from pre-runs determining the reasonable range of each parameter (Table 3). Twenty experiments (Table 4) were generated using CCD to study the effect of input (process) parameters on the surface roughness of drawpieces. However, unsuccessful runs were eliminated from the ANOVA analysis due to insufficient size of the formed height causing an inability to obtain comparable results.

**Figure 3:**
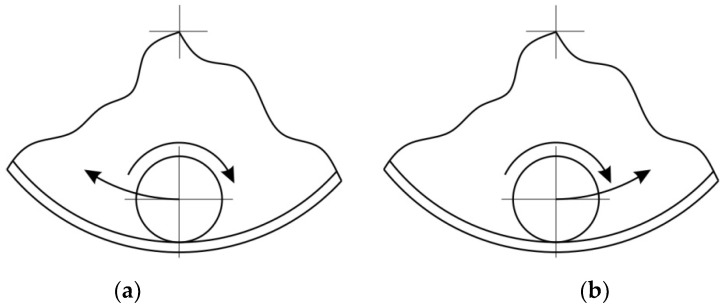
Toolpaths strategies: (**a**) conventional and (**b**) climb.

If the tool and the toolpath are both moving clockwise or counterclockwise, it is climb milling, and if they are rotating in different directions, it is up milling [67]. When the tool and the toolpath move in opposite directions (Figure 3a), there is a more intense friction interaction between the tool and sheet metal. The toolpath conventional strategy (Figure 3a) is the most commonly used mode in SPIF, as the friction is reduced by the tool effectively ‘rolling’ over the sheet as it forms [68]. Switching of the rotation direction from conventional to climbing was made by changing the feed direction (Figure 3b).

### 2.5. Surface Characteristics

**Figure 4:**
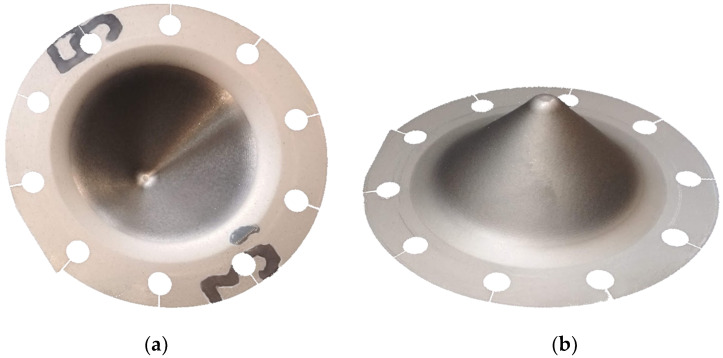
Successfully formed specimen: (**a**) inner side (**b**) outer side.

The following surface parameters were measured inside and outside the specimen surface (Figure 4): skewness (Ssk), kurtosis (Sku), maximum height (Sz), arithmetical mean height (Sa), root mean square gradient (Sdq), and reduced peak height (Spk).

Skewness (S_sk_) parameter characterizes the surface height asymmetry distribution, it describes the degree of bias of the roughness shape [69]:

(1) $$\mathrm{Ssk}=\frac{1}{S_{q}^{3}}\frac{1}{\mathrm{MN}}\overset{N}{\underset{j=1}{\sum }}\overset{M}{\underset{i=1}{\sum }}\eta^{3}\left(x_{i},{\;y}_{j}\right)$$

where M, N are the length and width of the given section of surface corresponding to the baseline for the given type of surface irregularities, η (x_i_, y_j_) is the deviation of the surface irregularities from the base plane.

Kurtosis (Sku) characterises the surface height distribution aspect ratio. Sku parameter is a gauge of the sharpness of the profile roughness [69]:

(2) $$\mathrm{Sku}=\frac{1}{S_{q}^{4}}\frac{1}{\mathrm{MN}}\overset{N}{\underset{j=1}{\sum }}\overset{M}{\underset{i=1}{\sum }}\eta^{4}\left(x_{i},{\;y}_{j}\right)$$

Maximum height (Sz) is the mean value of the absolute heights of the five highest peaks and the five lowest depressions within the sampling area, where: _v_η_vj_ (i = 1, 2, 3, 4, 5) denotes the five highest peaks and the five lowest pits in the sampling area, respectively [69]:

(3) $$\mathrm{Sz}=\frac{\sum_{i=1}^{5}\left|\eta_{p_{i}}\right|-\sum_{j=1}^{5}\left|\eta_{v_{j}}\right|}{5}$$

Arithmetical mean height (Sa) is the arithmetic mean value of the residual surface roughness deviation within the sample area. Expressed as the absolute value of the difference in height of each point compared with the arithmetic mean of the area [69]:

(4) $$\mathrm{Sa}=\frac{1}{\mathrm{MN}}\overset{N}{\underset{j=1}{\sum }}\overset{M}{\underset{i=1}{\sum }}\left|\eta \left(x_{i},{\;y}_{j}\right)\right|$$

Root mean square gradient (Sdq) calculates the local slope in each triangle of the surface mesh, where $\rho_{\mathrm{ij}}^{2}$ is the slope in the given surface coordinates [69]:

(5) $$\mathrm{Sdq}=\sqrt{\frac{1}{\left(M-1\right)\left(N-1\right)}\overset{N}{\underset{j=2}{\sum }}\overset{M}{\underset{i=2}{\sum }}\rho_{\mathrm{ij}}^{2}}$$

Reduced peak height (Spk) describes the mean height of the peaks above the surface core. A large Spk informs that a surface with high peaks provides a small initial contact area and thus large contact stress areas when in contact with the surface [69]:

(6) $$\mathrm{Spk}=\eta_{\max}-\eta_{1}$$

The 3D surface roughness parameters were measured using a Talysurf CCI Lite white light interferometer with a vertical resolution of 0.01 nm. Measurements were conducted according to the ISO 25178-2 [69] standard. Surface morphology of the inner surface of drawpieces was examined using an S-3400 Phenom ProX scanning electron microscope (SEM). Surface roughness parameters were measured on successfully formed drawpieces with a height of h = 28.3 mm (Figure 2). The surface roughness parameters were measured on an area of 2.5 × 3.0 mm at a location at half the height of the drawpieces.

### 2.6. Artificial Neural Networks

Artificial neural networks are tools that enable nonlinear models that solve complex classification and regression tasks to be constructed. Calculations performed by neural networks belong to the group of so-called soft computing processes [70]. The structure and the essence of the operation of ANNs is a reflection of the biological brain. They are a set of interconnected elements called neurons that process information delivered to the input of the network based on the idea of parallel processing.

**Figure 5:**
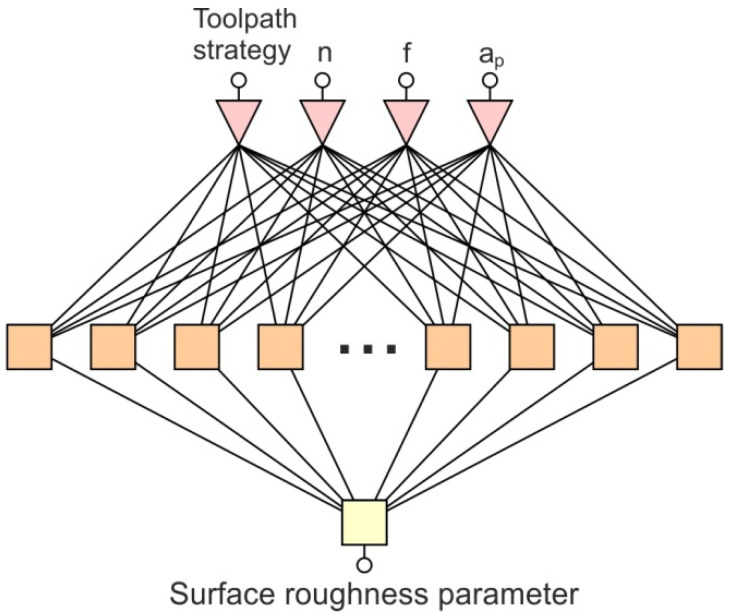
Architecture of a multilayer perceptron: n—spindle speed, f—feed rate, a_p_—incremental step size.

In this article, ANNs are used to define the process parameters that significantly affect the value of the specific roughness parameters measured on the inner and outer sites of the drawpieces. The Statistica program, which contains a module for neural calculations, was used for the analysis. This is a tool that allows the data entered to be automatically analysed and a set of networks of the best quality to be determined. In general, the multilayer network (multilayer perceptron–MLP) architecture consists of an input layer, a hidden layer, and an output layer (Figure 5). The values of the SPIF parameters were presented in the input layer. On the other hand, the value of a specific roughness parameter was presented to the network as an output parameter. While the number of input and output neurons is determined by the number of variables introduced to the input, the selection of the number of neurons in this hidden layer is a complicated task. Many network architectures were tested to obtain the network with the lowest mean square error RMS:

(7) $$\mathrm{RMS}=\sqrt{\frac{\sum_{i=1}^{N}{\left(z_{i}-y_{i}\right)}^{2}}{N}}$$

where: N—number of vectors in the training set, y_i_—output signal for i-th data set, z_i_—expected signal for i-th data set.

For each roughness parameter in the output layer, an independent network architecture was built to ensure the lowest RMS error value. For training the network, the back propagation algorithm was used, which is one of the most effective algorithms for training multilayer networks [71]. The data of successfully formed drawpieces (h = 28.3 mm according to Table 4) were used as the training set. To ensure the correct operation of the training algorithm, 10% of the data were separated from the entire training set and assigned to the validation set as suggested in papers [72,73]. Data from this subset are used to independently check the convergence of the training algorithm.

Correct operation of the neural network requires the transformation of the original data (normalisation) through their scaling to a small interval. The most useful intervals when analysing issues related to neural networks and data mining are 〈−1,+1〉 [74]. The min − max normalisation method was used, which uses a linear function to transform the raw data values into a new interval (D_min_, D_max_):

(8) $$D^{\prime }=\frac{\left(D-\min\right)}{\max-\min}\left(D_{\max}-D_{\min}\right)+D_{\min}$$

where D—value of the variable subjected to normalization, (min, max) is the interval in which the original data are contained.

The toolpath strategy is decoded as a nominal two-state non-numeric variable.

### 2.7. Analysis of Variance

An analysis of variance was performed to understand the adequacy of the proposed model and the influence of the input parameters on the specific roughness parameter. The analyses were performed for all surface roughness parameters considered in the ANN modelling. As the process parameters potentially influenced the specific roughness parameter, the following SPIF parameters were considered: step size a_p_, feed rate f, spindle speed n, and toolpath strategy (direction of tool rotation with regard to feed direction).

The analysis of variance was performed in the DesignExpert program for statistical analyses. The F-test was used to verify the quality of the regression model, and the multiple comparison procedure taking into account the statistical significance of the dependent variable was based on F-statistics at the significance level α = 0.05. The significance of the input variables was determined by the probability of *p* = 0.1000.

## 3. Results and Discussion

### 3.1. Surface Roughness

Surface roughness parameters of the inner and outer surface of the drawpieces are shown in Table 5 and Table 6, respectively. The values of all examined surface roughness parameters are provided for information purposes. A change in surface roughness parameters is hard to interpret; therefore, artificial neural networks and ANOVA were used for this purpose. The results of these analyses will be presented in the following subsections.

**Figure 6:**
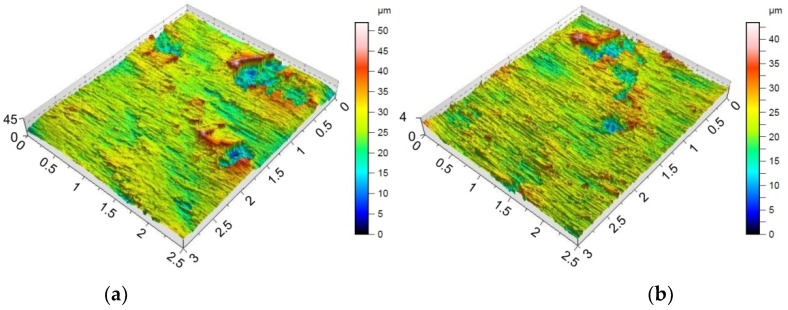
Topography of inside surface of the drawpieces formed at a_p_ = 0.3 mm, f = 1250 mm/min, *n* = 789.64 rpm and toolpath strategy: (**a**) conventional and (**b**) climb.

**Figure 7:**
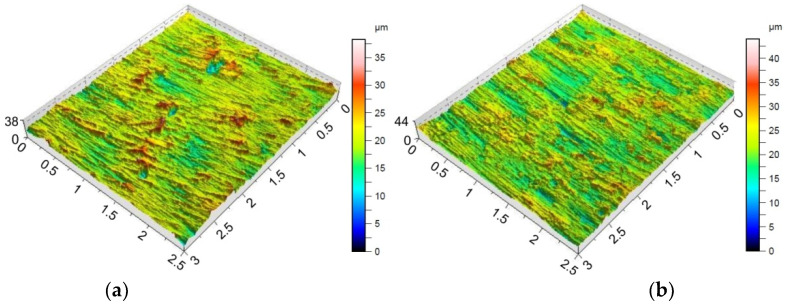
Topography of inside surface of the drawpieces formed at a_p_ = 0.3 mm, f = 1250 mm/min, *n* = 400 rpm and toolpath strategy: (**a**) conventional and (**b**) climb.

**Figure 8:**
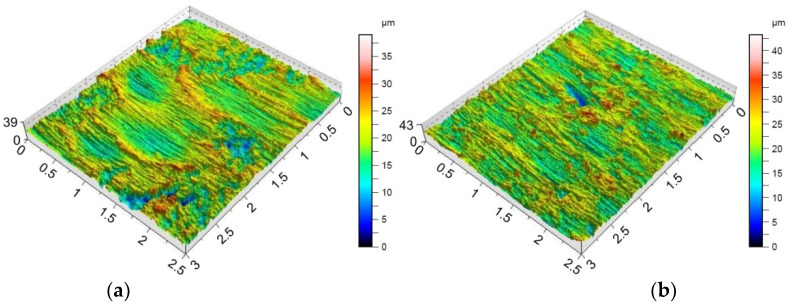
Topography of inside surface of the drawpieces formed at a_p_ = 0.5 mm, f = 500 mm/min, *n* = 600 rpm and toolpath strategy: (**a**) conventional and (**b**) climb.

**Figure 9:**
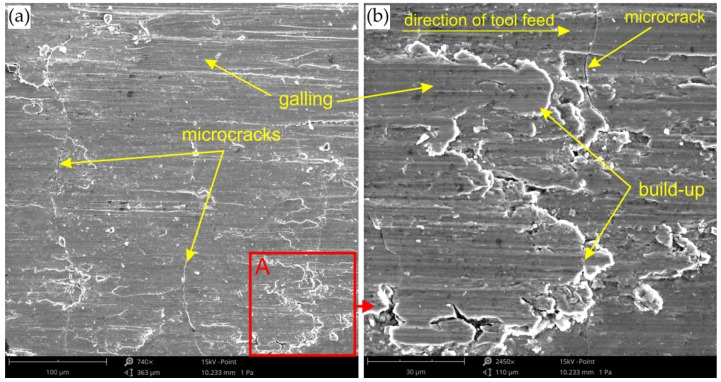
(**a**) SEM micrograph of the inner surface of the drawpiece formed with a_p_ = 0.1 mm, f = 2000 mm/min and *n* = 600 rpm, (**b**) magnification of the A area.

**Figure 10:**
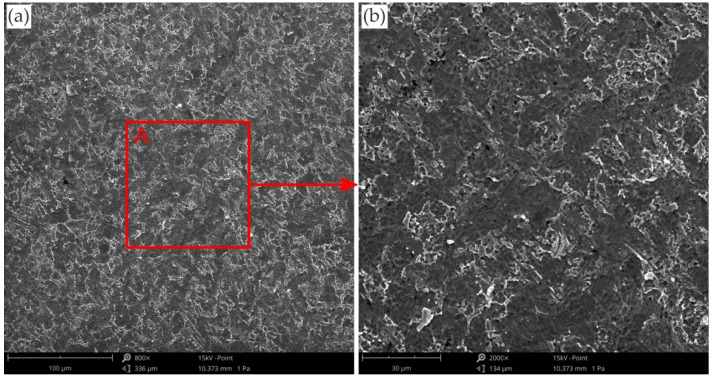
(**a**) SEM micrograph of the outer surface of the drawpiece formed with a_p_ = 0.1 mm, f = 2000 mm/min and *n* = 600 rpm, (**b**) magnification of the A area.

Providing appropriate conditions is particularly important during SPIF, in which the pin tool locally exerts very high pressure on the sheet material and causes an increase in temperature in the contact zone. Inadequate frictional conditions can lead to severe damage on the inner surface of the drawpiece. Figure 6, Figure 7 and Figure 8 shows the surface morphology of the inner surfaces of the drawpieces. Machining marks left by the tool are visible on these surfaces. During forming with conventional strategy (Figure 3a), the tool interacts more severely than with the toolpath climb strategy (Figure 3b). Although areas with a more differentiated surface profile height are visible in the toolpath conventional strategy (Figure 6a, Figure 7a, Figure 8a) compared to those formed with the toolpath climb strategy (Figure 6b, Figure 7b, Figure 8b), the height of the surface profile is comparable for both strategies. In the toolpath conventional strategy conditions, a more intensive method of interaction between the tooltip and the sheet surface were observed. The outer surface of the drawpiece is subjected to localised tensile and bending loads during SPIF [75], resulting in tensile stresses on the surface, potentially causing sheet surface defects (including voids, microcracks, and orange peel). Reducing the rotational speed from 789.64 rpm (Figure 6) to 400 rpm (Figure 7), with the same feed rate f = 1250 mm/min and step size ap = 0.3 mm, reduced the effect of intense seizing of the sheet surface during forming with both toolpath strategies. The defect density and the height of the surface profile increase with the deformation value, and as a consequence, can lower the fatigue strength of the products [76]. Microcracks (Figure 9a,b) oriented perpendicularly to the direction of the tool feed appeared on the inner surface of the drawpiece. The sheet surface is strongly work-hardened by plastic deformation and loses its elastic properties. During forming at the point of contact of the tool with the sheet metal, multiple elastic deformations of the sheet material occur. As a result of cyclic deformations and springback phenomena, the surface of the sheet becomes susceptible to cracking. Microcracks in the sheet surface may also occur, especially when forming materials that are difficult to deform and strongly work-hardened [76]. The outer surface of the drawpieces was very rough with an orange peel character (Figure 10a,b). The change in surface roughness is clearly related to the grain microstructure of the material. Plastic deformation of the material surface produced small dimples which, with sufficiently high plastic deformation, are a symptom of ductile fracture formation.

**Figure 11:**
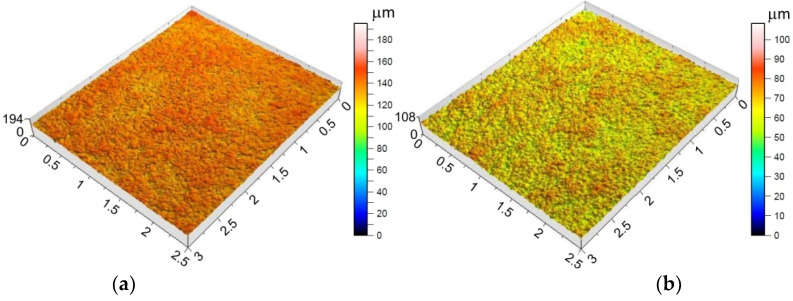
Topography of the outside surface of the drawpieces formed at a_p_ = 0.3 mm, f = 1250 mm/min, *n* = 789.64 rpm and toolpath strategy: (**a**) conventional and (**b**) climb.

**Figure 12:**
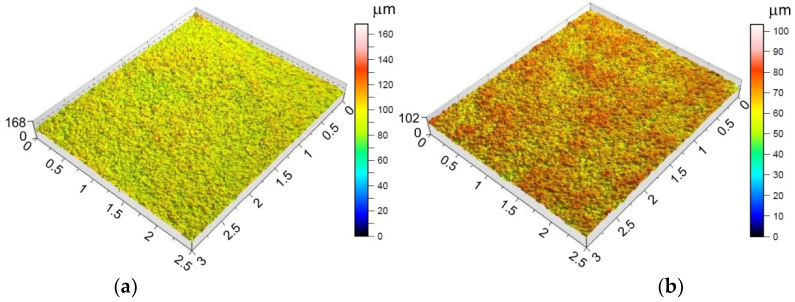
Topography of the outside surface of the drawpieces formed at a_p_ = 0.5 mm, f = 500 mm/min, *n* = 600 rpm and toolpath strategy: (**a**) conventional and (**b**) climb.

The morphology of the outer surface of the drawpieces (Figure 11 and Figure 12) is uniform with a much greater profile height than the inner surface that has interacted with the tool. A clear orange peel effect was observed for all variants of the processing parameters. Orange peel occurs when the tool contacts the sheet on one side only and is a result of the different orientations of the adjacent grains on the surface [77]. During the gradual deformation of different values along the circumference of the drawpiece and along the cone generatrix, they tend to thin or thicken in different ways. In the context of this, Hamilton and Jeswiet [78] found that the most sensitive element for the extent of orange peel formation was the ratio between the step size and forming angle. Due to the more intensive operation of the tooltip in the toolpath conventional strategy, the outer surface also has an increased profile (Figure 11a and Figure 12a) compared to the toolpath climb strategy (Figure 11b and Figure 12b). The more intense interaction of the tooltip with the inner surface of the drawpiece causes a higher temperature through the sheet metal so that the material is more susceptible to deformation. This phenomenon is exploited in the friction-assisted variants of SPIF used to form difficult-to-deform materials [79].

### 3.2. Artificial Neural Networks

The results of the sensitivity analysis of the effect of individual process parameters on the value of specific surface roughness parameters are shown in Table 7 and Table 8. Due to the difficulty to define a priori interactions between parameters and the various influences of input parameters on the specific surface roughness parameter, an independent network was built for each parameter (‘ANN structure’ column).

In the case of the Sdq and Spk parameters measured on the inner surface (Table 7) and the outer surface (Table 8), the parameter that is most correlated with these parameters is the toolpath strategy. In contrast, tool rotational speed shows the lowest information capacity. Taking into account the outer surface of the drawpiece, the toolpath strategy is also most closely correlated with the parameters Sa, Sz, and Sku. Only in the case of skewness can the toolpath strategy be considered as the least significant parameter. It is interesting that the ‘Rank’ of the input parameters that are considered has affected the Sa, Sdq, and Spk parameters in the same way (Table 8). The influence of the toolpath strategy and step size on the Sz parameter measured at the inner (Table 7) and outer (Table 8) surface of the drawpieces is inverse. The toolpath strategy is the most significant in the case of the inner side of the drawpiece while the ‘Rank’ of step size is the least important. In the case of the outer surface of the drawpiece, the situation is the reverse (Table 8). Hagan and Jeswiet [80] found that due to the sinusoidal-type profile across the tool path, it is more useful to assess the inner surfaces of drawpieces using the Rz parameter. The information capacity of the input parameters on the skewness and kurtosis measured on the inner surface of drawpieces is quite similar (Table 7). Step size and feed rate provide the largest information capacity in relation to the skewness and kurtosis of the inner surface of the drawpiece. The toolpath strategy has the least effect on the maximum height Sz, which was also noticed earlier on the basis of the topographies of inner surfaces shown in Figure 6, Figure 7 and Figure 8. The profile height was similar for both strategies considered.

The quality of the neural network is assessed on the basis of the standard deviation (SD) ratio and the Pearson’s r correlation coefficient. The ‘SD ratio’ parameter is the standard deviation of the errors and standard deviation of the real data. The data statistics selected for all networks listed in Table 7 and Table 8 are presented in Table 9. For a very good model, the value of the standard deviation ratio is below 0.1. A high value of the Pearson’s r correlation measure close to R = 1 with a low value of the SD ratio proves the good approximation capabilities of the neural networks. Only one network has a Pearson’s r correlation value below 0.9.

### 3.3. ANOVA

Analysis of variance was performed to understand the adequacy of the proposed model and the influence of the input parameters on the specific roughness parameter. The analyses were performed for all surface roughness parameters considered in the ANN modelling. DesignExpert conducted an automatic analysis of the data and suggested ‘the best’ statistical model. Table 10 shows the comparison of the statistics of regression models suggested by the DesignExpert program.

The signal-to-noise ratio is represented by the adequacy precision parameter. A value lower than four indicates an inadequate signal. In this regard the regression models for Ssk, Spk measured in the inner side of the drawpiece, and for Ssk, Sku, Sz, Sdq, Spk parameters measured on the outer side of the drawpieces cannot be used to navigate the design space. Moreover, the F-value of these models implies that they are not significant relative to the noise. For example, considering the Ssk surface roughness parameter measured on the inner side of the drawpiece there is a 37.62% chance that an F-value this large could occur due to noise. When considering the R^2^-value of the model, the SPIF parameters are better correlated with the roughness parameters measured on the inner side of the drawpiece. The model F-value and adequacy precision imply the models for Sa, Sdq and Sz measured on the inner side of the drawpiece, and the Sa parameter measured on the outer side of drawpieces can be used to navigate the design space. There is only between a 0.13% (Sdq) and 4.82% (Sa) chance that an F-value this large could occur due to noise. P-values less than 0.5 imply the models are significant. The detailed fit statistics for the significant ANOVA models marked in Table 10 are listed in Appendix A. In the case of the Sa parameter measured on the inner surface of the drawpiece, the feed rate is the most significant parameter (Table A1). However, spindle speed affects the value of the Sa parameter measured on the outer surface of the drawpiece the most (Table A4). The spindle speed and step size have the least influence on the Sdq value of the inner surface of the drawpiece (Table A2) which is in agreement with the results of the sensitivity analysis carried out using ANNs (Table 7). The direction of tool rotation, followed by feed rate, has the smallest effect on the Sz parameter of the inner side of the drawpiece (Table A3) which also corresponded with the ANN results (Table 7).

**Figure 13:**
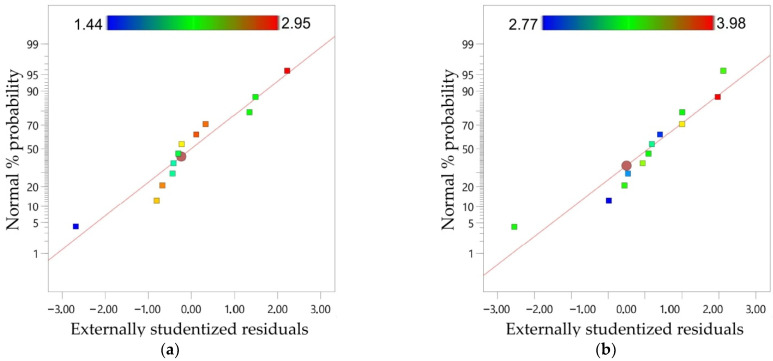
Normal probability plot of residuals for the Sa parameter measured on the (**a**) inner and (**b**) outer side of the drawpiece.

The ANOVA model was only significant for arithmetical mean height Sa measured on both inner and outer surfaces of the drawpiece. So, the results for the Sa parameter will be presented in more detail below. The proportional distribution of normal probability along the straight line observed for both models (Figure 13a,b) proves that the model errors have a normal distribution [81]. This is a condition for a good correlation between actual and predicted values and for statistical significance of the whole regression model.

**Figure 14:**
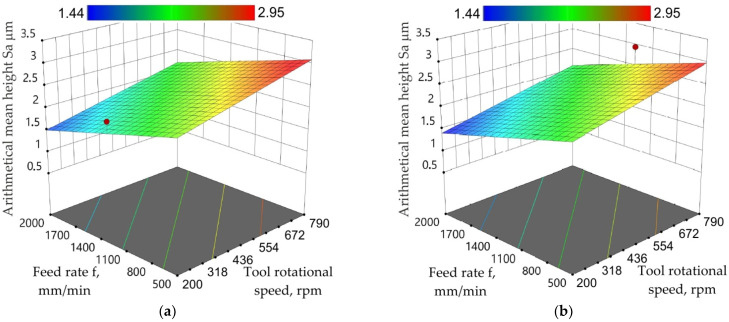
Response surface plots presenting the interaction between feed rate and tool rotational speed affecting the arithmetical mean height Sa measured on the inner side of the drawpiece for a_p_ = 0.3 mm and toolpath (**a**) conventional and (**b**) climb strategies.

In the entire range of feed rate changes, increasing the tool rotational speed increases the value of the Sa parameter (Figure 14a,b). The character of the response surface changes for arithmetical mean height is very similar for both toolpath strategies. The decrease in the Sa parameter with the reduction of tool rotational speed (Figure 14a) was also observed by Trzepieciński et al. [58]. The maximum surface finish can be obtained with a higher feed rate and minimum tool rotational speed (Figure 14b) [82].

**Figure 15:**
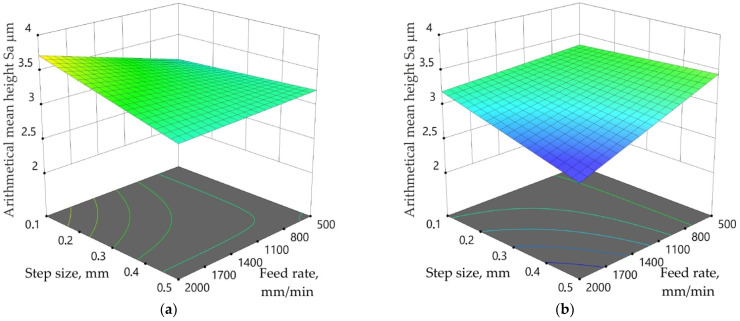
Response surface plots presenting the interaction between step size and feed rate affecting the arithmetical mean height Sa measured on outer surface of the drawpiece for *n* = 495 rpm and toolpath (**a**) conventional and (**b**) climb strategies.

Different dependencies between input parameters measured on the outer surface of the drawpiece were found for the toolpath strategies analysed (Figure 15a,b). In the case of the toolpath conventional strategy, for the largest step size value examined (a_p_ = 0.5 mm) changing the feed rate does not affect the value of the Sa parameter. The same relationship was observed for the toolpath climb strategy; however, for the smallest step size value (a_p_ = 0.1 mm). The lowest value of the Sa parameter on the outer surface of the drawpiece SPIFed with the toolpath climb strategy was obtained for the highest values of both feed rate and tool rotational speed (Figure 15b). Feed rate plays a key role in the difference in the Sa parameter of the surface of the drawpiece formed by the different toolpath strategies. When forming with a small step size value (a_p_ = 0.1 mm) in the toolpath conventional strategy, a clear increase in Sa-value was observed with an increase in feed rate (Figure 11a). A different relationship exists between the toolpath climb strategy and the highest step size considered (Figure 15b). The difference is due to the different actions of the tool on the sheet metal. The toolpath conventional strategy causes a more severe interaction of the tool and sheet metal compared to the climb strategy. In this way, the generation of frictional heat improves the formability of the workpiece material. Forming with the toolpath climb strategy is gentler and with suitably small step size, no influence of the feed rate on the Sa parameter could be found (Figure 15b). With the minimum step size, the overlap of the form tool between the two successive tool paths reduces the peaks and valleys on the formed surface, thus improving the surface finish [82].

## 4. Conclusions

This article presents the results of the analysis of the influence of the SPIF parameters (step size, feed rate, and tool rotational speed) on the surface roughness measured on both sides of conical drawpieces made from pure titanium Grade 2 sheets. Both toolpath conventional and toolpath climb strategies were analysed. Based on the results, the following main conclusions can be drawn:

- Although there are visible areas with a more differentiated surface profile height in the toolpath conventional strategy compared to those forming with the toolpath climb strategy, the height of the surface profile height is comparable for both strategies.
- Cracks observed on the inner side of the drawpiece are a result of the intensive strain hardening of the inner subsurface layer and cyclic elastic deformation of the workpiece material during SPIF.
- The orange peel effect related to the increase in profile height was observed on the outer surface of drawpieces.
- Step size and feed rate provide the highest information capacity in relation to skewness and kurtosis of the inner surface of the drawpiece.
- Increasing the profile height was greater during SPIF with the conventional strategy compared to the toolpath climb strategy.
- Taking into account the outer surface of the drawpiece, the direction of tool rotation is most closely correlated with the parameters Sa, Sz, and Sku.
- In the case of the inner side of the drawpiece, the toolpath strategy is the most correlated with the Sz parameter.
- In the entire range of feed rate changes, increasing the tool rotational speed increases the value of the Sa parameter.

The use of an experimental plan created on the basis of central composite design and analysis of the sensitivity of the SPIF process parameters on the selected surface roughness parameters using artificial neural networks will allow reducing the time of introducing the new product in the industry. With the knowledge about the influence of SPIF parameters on the surface topography of the inner and outer surfaces of conical drawpieces, we can choose one or more representative roughness parameters to assess the quality of components on both sides. The outer surface of the drawpieces changes significantly, mainly due to the ‘orange peel’ effect, and its quality also needs to be controlled. One of the justifications is to prepare the surface for painting, which must have the appropriate roughness.

## Figures and Tables

**Table 1:** Chemical composition of the CP Grade 2 titanium sheet selected (in weight%).

C	N	O	Fe	Ti
0.009	0.009	0.23	0.12	balance

**Table 2:** Basic mechanical properties of CP grade 2 titanium sheet.

YS, MPa	UTS, MPa	SHC, MPa	SHE
273 MPa	359 MPa	655 MPa	0.137

**Table 3:** The range of experiments presented by the input factors.

Input Parameter	Low Level	High Level
Step size a_p_, mm	0.1	0.5
Feed rate f, mm/min	500	2000
Spindle speed n, rpm	−600	600

**Table 4:** Central composite design of experiments.

Number of Experiment	Incremental Step Size a_p_, mm	Feed Rate f, mm/min	Spindle Speed n, rpm	Height of Drawpiece h, mm
1	0.3	262.94	0	5.6
2	0.3	1250	−200	28.3
3	0.1	2000	−600	28.3
4	0.1	2000	600	8.6
5	0.3	1250	−789.64	28.3
6	0.3	2237.06	200	7.9
7	0.5	500	600	28.3
8	0.3	1250	−400	28.3
9	0.3	1250	0	6.6
10	0.1	500	600	28.3
11	0.563	1250	0	7.6
12	0.1	500	−600	28.3
13	0.3	1250	789.64	28.3
14	0.5	2000	−600	28.3
15	0.3	1250	0	5.9
16	0.3	1250	200	6.8
17	0.5	2000	600	28.3
18	0.036	1250	0	5.5
19	0.5	500	−600	28.3
20	0.3	1250	400	28.3

**Table 5:** Surface roughness parameters measured on the inner surface of the drawpieces.

Run No.	Ssk	Sku	Sz, µm	Sa, µm	Sdq	Spk, µm
2	0.347	3.91	33.4	2.2	0.318	3.22
3	−1.13	8.93	42.3	1.94	0.245	2.98
5	−0.151	5.95	43.4	2.59	0.301	4.04
7	−0.138	3.51	39	2.79	0.3	3.11
8	0.215	3.96	44.1	2.1	0.299	2.81
10	0.063	3.78	48.3	2.83	0.306	3.22
12	−0.035	5.1	50.4	2.75	0.325	3.69
13	0.295	5.42	45.2	2.95	0.272	5.65
14	0.231	4.85	43.7	2.21	0.28	3.59
17	−0.162	3.33	34.4	1.44	0.199	1.27
19	0.152	3.86	43.3	2.65	0.339	3.63
20	0.51	5.07	38.3	1.98	0.258	3.99

**Table 6:** Surface roughness parameters measured on the outer surface of the drawpieces.

Run No.	Ssk	Sku	Sz, µm	Sa, µm	Sdq	Spk, µm
2	−0.186	3.85	74.3	2.77	0.473	2.84
3	−0.195	8.54	136	3.98	1.01	4.15
5	−0.47	6.22	108	3.4	0.657	3.35
7	−0.294	15.3	168	3.7	1.19	4.82
8	−1.29	37.5	239	3.21	1.17	4.35
10	−0.199	4.47	100	3.42	0.622	2.88
12	−0.396	8.43	142	3.45	0.835	3.3
13	−0.447	13.6	195	3.55	0.91	4.03
14	−0.455	19.5	149	3.38	1.18	4.36
17	−0.369	4.74	74	2.83	0.542	2.68
19	−0.239	4.42	103	3.38	0.622	3.24
20	−0.306	4.61	74.8	2.95	0.592	2.89

**Table 7:** The results of the sensitivity analysis for surface roughness parameters measured on the inner side of the drawpiece.

Surface Roughness Parameter	ANN Structure	Parameter	Toolpath Strategy	*n*	f	a_p_
Ssk	4:4-7-1:1	Rank	3	4	2	1
		Error	0.165	0.110	0.168	0.226
		Ratio	1.910	1.275	1.950	2.610
Sku	4:4-10-1:1	Rank	3	4	1	2
		Error	0.104	0.102	0.218	0.178
		Ratio	2.941	2.882	6.174	5.023
Sz	4:4-10-1:1	Rank	4	2	3	1
		Error	0.133	0.219	0.203	0.225
		Ratio	0.911	1.498	1.390	1.535
Sa	4:4-8-1:1	Rank	2	3	1	4
		Error	0.213	0.173	0.266	0.065
		Ratio	3.784	3.078	4.729	1.159
Sdq	4:4-9-1:1	Rank	1	4	2	3
		Error	0.258	0.051	0.178	0.057
		Ratio	5.481	1.092	3.796	1.181
Spk	4:4-12-1:1	Rank	1	4	3	2
		Error	0.346	0.101	0.103	0.223
		Ratio	9.899	2.913	2.955	6.387

**Table 8:** The results of sensitivity analysis for surface roughness parameters measured on the outer side of the drawpiece.

Surface Roughness Parameter	ANN Structure	Parameter	Toolpath Strategy	*n*	f	a_p_
Ssk	4:4-7-1:1	Rank	4	1	3	2
		Error	0.021	0.070	0.062	0.065
		Ratio	1.170	3.043	2.700	2.834
Sku	4:4-8-1:1	Rank	1	2	4	3
		Error	0.474	0.349	0.163	0.214
		Ratio	4.959	3.653	1.706	2.245
Sz	4:4-14-1:1	Rank	1	2	3	4
		Error	0.595	0.493	0.443	0.196
		Ratio	8.708	7.216	9.491	2.878
Sa	4:4-13-1:1	Rank	3	4	1	2
		Error	0.109	0.105	0.236	0.137
		Ratio	1.406	1.356	3.046	1.769
Sdq	4:4-15-1:1	Rank	1	4	2	3
		Error	0.578	0.242	0.361	0.267
		Ratio	3.168	1.325	1.981	1.467
Spk	4:4-14-1:1	Rank	1	4	2	3
		Error	0.491	0.169	0.288	0.220
		Ratio	4.102	1.413	2.414	1.843

**Table 9:** Regression statistics of the ANNs analysed *.

Parameter	Ssk		Sku		Sz		Sa		Sdq		Spk	
	Inner Surface	Outer Surface	Inner Surface	Outer Surface	Inner Surface	Outer Surface	Inner Surface	Outer Surface	Inner Surface	Outer Surface	Inner Surface	Outer Surface
EM	−0.032	0.008	0.00003	−0.047	0.090	0.064	0.012	−0.004	−0.033	−0.031	0.004	−0.057
ESD	0.084	0.022	0.037	0.087	0.121	0.023	0.057	0.045	0.034	0.189	0.036	0.110
AE	0.074	0.020	0.023	0.055	0.126	0.064	0.052	0.028	0.040	0.124	0.027	0.102
SD ratio	0.304	0.235	0.131	0.279	0.379	0.068	0.179	0.150	0.163	0.478	0.147	0.312
R^2^	0.952	0.973	0.991	0.975	0.925	0.998	0.983	0.990	0.986	0.880	0.996	0.964

* EM—error mean, ESD—standard deviation of error, AE—average absolute error, SD ratio—data standard deviation ratio, R^2^—the standard Pearson’s r correlation.

**Table 10:** Fit statistics of the models considered.

Side of the Drawpiece	Output Variable	Sum of Squares	Mean Square	F-Value	*p*-Value	Assessment of the Model	R^2^	Adequacy Precision
inner	Ssk	0.7289	0.1822	1.06	0.4441	not significant	0.3762	3.2999
	Sku	18.30	4.58	4.07	0.0514	not significant	0.6993	7.1793
	Spk	2.46	0.6162	0.7944	0.7413	not significant	0.2203	1.9254
	**Sa**	**1.78**	**0.4448**	**5.71**	**0.0230**	**significant**	**0.7653**	**5.8899**
	**Sdq**	**0.0150**	**0.0037**	**15.91**	**0.0013**	**significant**	**0.9009**	**12.7038**
	**Sz**	**204.24**	**51.06**	**4.39**	**0.0433**	**significant**	**0.7149**	**5.9549**
outer	Ssk	0.0717	0.0179	0.1386	0.9626	not significant	0.0734	1.0717
	Sku	86.51	21.63	0.1568	0.9523	not significant	0.0822	1.2885
	Sz	2364.57	591.14	0.1526	0.9558	not significant	0.0802	1.2081
	Sdq	0.0795	0.0199	0.1964	0.9325	not significant	0.1009	1.2387
	Spk	0.7975	0.1994	0.2770	0.8839	not significant	0.1366	1.4098
	**Sa**	**1.17**	**0.1949**	**5.05**	**0.0482**	**significant**	**0.8583**	**7.1414**

## Data Availability

The data presented in this study are available on request from the corresponding author.

## Appendix A

**Table A1:** ANOVA results of the Sa parameter measured on the inner side of the drawpiece.

Source	Sum of Squares	Degrees of Freedom	Mean Square	F-Value	*p*-Value	Assessment of the Model
Model	1.78	4	0.4448	5.71	0.0230	significant
A—Spindle speed	0.4073	1	0.4073	5.23	0.0561	
B—Feed rate	1.26	1	1.26	16.19	0.0050	
C—Step size	0.0133	1	0.0133	0.1705	0.6921	
D—direction of tool rotation	0.0252	1	0.0252	0.3237	0.5872	
Residual	0.5455	7	0.0779			
Cor. Total	2.32	11				
Standard deviation	0.2792					
Mean	2.37					
Coefficient of variation %	11.78					

**Table A2:** ANOVA results of the Sdq parameter measured on the inner side of the drawpiece.

Source	Sum of Squares	Degrees of Freedom	Mean Square	F-Value	*p*-Value	Assessment of the Model
Model	0.0150	4	0.0037	15.91	0.0013	significant
A—Spindle speed	0.0000	1	0.0000	0.2074	0.6626	
B—Feed rate	0.0114	1	0.0114	48.71	0.0002	
C—Step size	0.0001	1	0.0001	0.3804	0.5569	
D—direction of tool rotation	0.0049	1	0.0049	20.72	0.0026	
Residual	0.0016	7	0.0002			
Cor. Total	0.0166	11				
Standard deviation	0.0153					
Mean	0.2868					
Coefficient of variation %	5.34					

**Table A3:** ANOVA results of the Sz parameter measured on the inner side of the drawpiece.

Source	Sum of Squares	Degrees of Freedom	Mean Square	F-Value	*p*-Value	Assessment of the Model
Model	204.24	4	51.06	4.39	0.0433	significant
A—Spindle speed	87.20	1	87.20	7.50	0.0290	
B—Feed rate	37.41	1	37.41	3.22	0.1160	
C—Step size	50.45	1	50.45	4.34	0.0758	
D—direction of tool rotation	21.45	1	21.45	1.84	0.2166	
Residual	81.43	7	11.63			
Cor. Total	285.67	11				
Standard deviation	3.41					
Mean	42.15					
Coefficient of variation %	8.09					

**Table A4:** ANOVA results of the Sa parameter measured on the outer side of the drawpiece.

Source	Sum of Squares	Degrees of Freedom	Mean Square	F-Value	*p*-Value	Assessment of the Model
Model	1.17	6	0.1949	5.05	0.0482	significant
A—Spindle speed	0.5390	1	0.5390	13.96	0.0135	
B—Feed rate	0.0159	1	0.0159	0.4111	0.5496	
C—Step size	0.0740	1	0.0740	1.92	0.2249	
D—direction of tool rotation	0.0530	1	0.0530	1.37	0.2941	
BC	0.1589	1	0.1589	4.11	0.0983	
BD	0.1986	1	0.1986	5.14	0.0727	
Residual	0.1931	5	0.1931			
Cor. Total	1.36	11				
Standard deviation	0.1965					
Mean	3.34					
Coefficient of variation %	5.89					
